# Comparison of cyclic fatigue resistance of original and counterfeit rotary instruments

**DOI:** 10.1186/1475-925X-13-67

**Published:** 2014-05-31

**Authors:** Huseyin Ertas, Ismail Davut Capar, Hakan Arslan, Ender Akan

**Affiliations:** 1 Department of Endodontics, Faculty of Dentistry, Izmir Katip Celebi University, Izmir 35620, Turkey; 2 Department of Endodontics, Faculty of Dentistry, Atatürk University, Izmir, Turkey; 3 Department of Prosthodontics, Faculty of Dentistry, Izmir Katip Celebi University, Izmir, Turkey

**Keywords:** Counterfeit, Cyclic fatigue, Dental, Nickel-titanium files

## Abstract

**Introduction:**

In recent years, with the advances in counterfeiting methods, counterfeit products have reached the dental market. The purpose of this study was to compare the cyclic fatigue resistance of original and counterfeit rotary root canal instruments.

**Materials and methods:**

The cyclic fatigue of original and counterfeit ProTaper F2 endodontic instruments was tested (n = 20) in 3 mm radius steel canals with a 60° angle of curvature. The number of cycles to fracture (NCF) was calculated, and the data were subjected to the Student’s t-test (α = 0.05).

**Results:**

The original instruments showed better cyclic fatigue resistance than the counterfeit ones (*p* < .001). The mean NCF was 483 ± 84 for the original files and 186 ± 86 for the counterfeit files.

**Conclusions:**

The cyclic fatigue resistance of the counterfeit instruments was very low. As a result, clinicians should be careful not to purchase counterfeit products.

## Introduction

Nickel-titanium (Ni-Ti) rotary instruments were introduced to the dental profession in the early 1990s, and today these instruments are commonly used among dentists. Over the years, many different rotary Ni-Ti instruments have been marketed.

It is well-known that the separation of Ni-Ti rotary instruments is a risk to the success of dental treatments [1]. In clinical practice, the fracture of Ni-Ti rotary instruments occurs via two different mechanisms: torsional fracture and flexural fatigue [2]. Torsional fracture occurs when part of the instrument binds to the dentin, while the file continues to rotate [3]. However, flexural fatigue fracture of the file occurs when the instrument rotates freely in a curvature, generating tension/compression cycles in the region of maximum flexure, until fracture occurs [1].

In recent years, with the advancement of counterfeiting methods, counterfeit products have reached the dental market. These imitation medical products present potential health risks for patients, because the quality and/or performance of these products are largely unknown. From 2001-2009, thirteen counterfeit medical devices were reported, and four of them were involved in surgical implants and dental filling material [4]. However, the performance of any counterfeit endodontic products have not yet been investigated.

The purpose of this in-vitro study was to compare the cyclic fatigue resistance of counterfeit and original rotary files. The null hypothesis is that there is no significant difference between counterfeit and original rotary NiTi files with regard to cyclic fatigue resistance.

## Materials and methods

**Figure 1:**
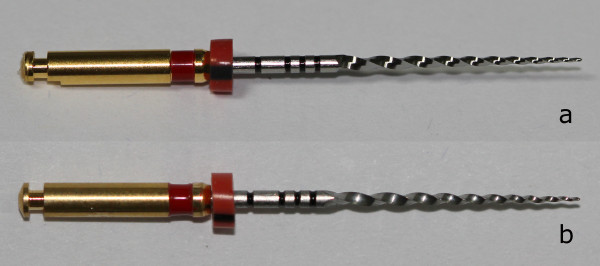
**Photographs of original (a) and counterfeit (b) rotary files.***Note the similarities between two files in macro photographs.*

**Figure 2:**
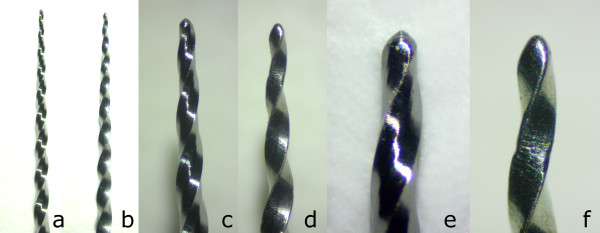
**Stereomicroscopic images of original (a, c, e) and counterfeit (b, d, f) products at 10 X (a, b), 25 X (c, d), and 50 X (e, f) magnification.***Note that the differences between the files could be seen under magnifications.*

The cyclic fatigue of the original ProTaper Universal files (Dentsply, Maillefer, Ballaigues, Switzerland) and counterfeit ones were tested (Figures 1 and 2). Twenty instruments from each group were evaluated in air at a temperature of 23°C. An artificial canal was made out of a testing block of stainless steel with an inner diameter of 1.5 mm, a 60° angle of curvature, and a curvature radius of 3 mm. The canals were covered with glass to prevent the instruments from slipping out. The apparatus used in the cyclic fatigue test was described previously (5). The insertion depth was standardized to 19 mm for all the files. To reduce the friction of the file as it contacted the artificial canal walls, a special oil (WD-40 Company, Milton Keynes, England) was used for lubrication. All the instruments were operated with a low-torque motor, 6:1 reduction handpiece (VDW Silver; VDW, Munich, Germany) and rotated with ProTaper Universal F2 files own mode (250 rpm and 200 g/cm torque). The instruments were used until fracture occurred, and the time to fracture was recorded in seconds. The number of cycles to fracture (NCF) was calculated using the following formula:

$$\text{NCF}=\text{time}\;\left(\text{seconds}\right)\;\mathrm{to}\;\text{failure}\times \text{rotational}\;\text{speed}/60$$

**Figure 3:**
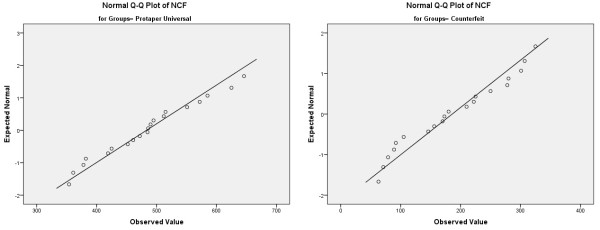
Q-Q plots of normally distributed data.

The broken instruments were ultrasonically cleaned in alcohol for scanning electron microscopy (SEM) examination. To verify the fracture mode, the surface of the fractured part of the samples from each group was photographed under SEM (Evo LS10; Carl Zeiss, Oberkochen, Germany).After completing the cyclic fatigue test, the NCF data were subjected to a Shapiro–Wilk test to analyze the normality of the continuous variables. Shapiro–Wilk test revealed that the distribution of the data (Figure 3) were normal (p > 0.05). The data were then statistically analyzed using Student’s t-test (α = 0.05).

## Results

**Figure 4:**
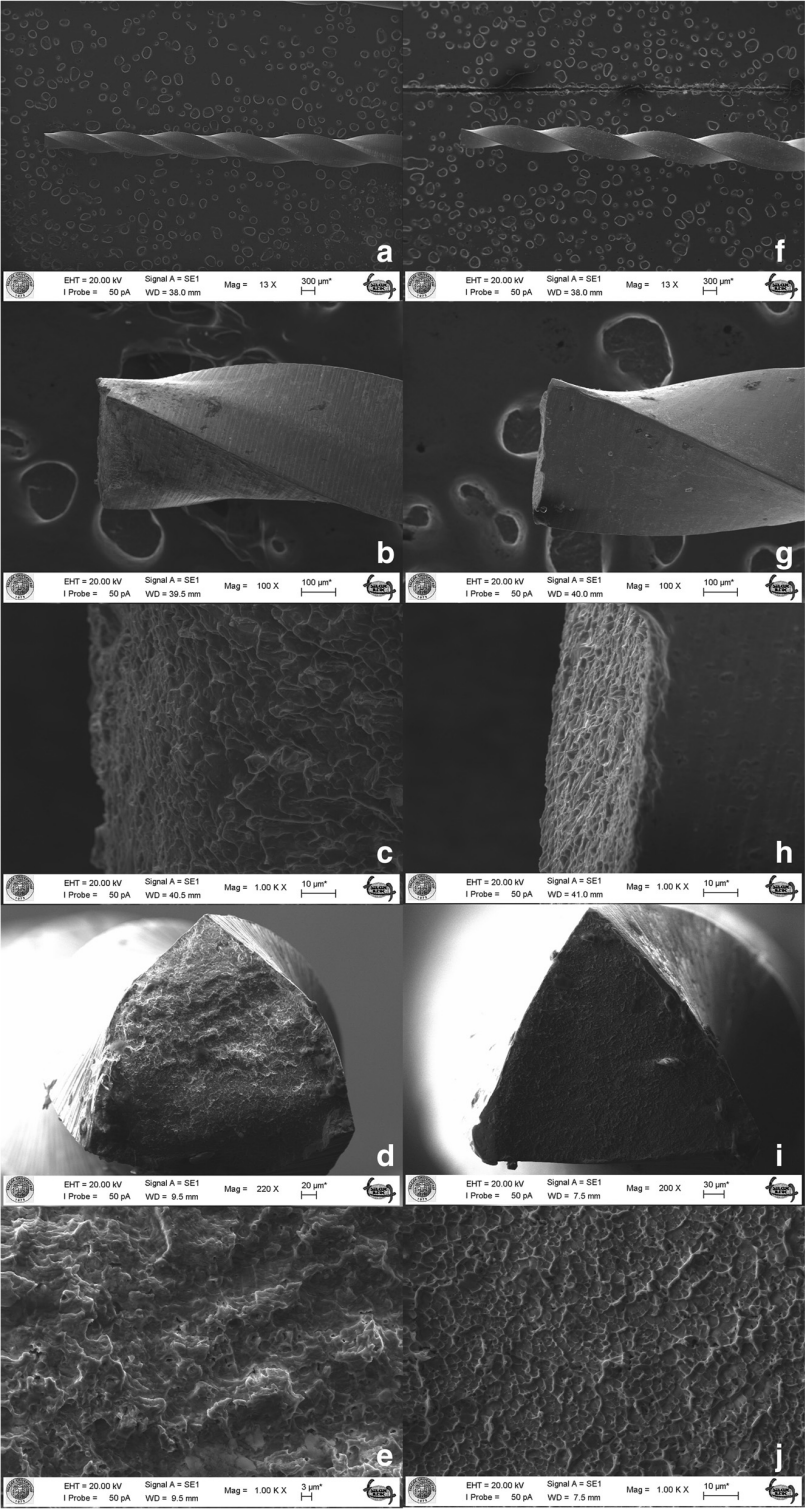
**Representative SEM examination of fractured instruments.** Left column **(a-e)** shows original, and right column **(f-j)** shows counterfeit products of rotary files. First and second line shows low and high magnification of lateral view of instruments respectively. The remaining three lines shows fracture surfaces of the test instruments. Despite the similarities between instruments, differences in cross sectional design, and morphology characteristics of fracture surface could be observed from the SEM images. The characteristic surface pattern with dimples, and cones produced by ductile rupture are observed at the fracture plane with **(e)** the ProTaper Universal and **(j)** the counterfeit one. Figure e/d comparison shows the fracture surface of a counterfeit instrument with much shallower dimples, which indicate less plastic deformation. Absence of circular abrasion marks indicates the flexural fatigue failure.

The NCFs of the fractured fragments for the groups are presented in Table 1. The original instruments presented significantly superior cyclic fatigue resistance when compared to the counterfeit instruments (*p* < .001). The percentage of standard deviation for the counterfeit group was higher than the original file group.The SEM images of the original file’s fracture surface show the nature of the mechanical damage of the cyclic fatigue failure. However, the cross section of the counterfeit file appeared to be different than the original one (Figure 2). The characteristic surface pattern with dimples, and cones produced by ductile rupture were observed at the fracture plane with (Figure 4e) the ProTaper Universal and (Figure 4j) the counterfeit one. Figure 4e,j comparison showed the fracture surface of a counterfeit instrument with much shallower dimples, which indicate less plastic deformation. Absence of circular abrasion marks on SEM images indicated the flexural fatigue failure.

**Table 1:** Comparison of mean NCF values of original and counterfeit files

**Groups**	**NCF**	**Min-Max**
Original	483±84	354-646
Counterfeit	186±86	63-325

## Discussion

In the present study, original and counterfeit rotary Ni-Ti instruments were tested. The findings of our study revealed that cyclic fatigue in the instruments, although having similar shapes, is quite different, and the quality of the imitation/counterfeit products is poor. Thus, the null hypothesis was rejected. The differences between the original and counterfeit instruments in their manufacturing processes and their alloys could have influenced the fatigue resistance of the instruments [5]. In addition, the cross-sections of counterfeit instruments were different from the original ones. Therefore, the results of this study could be influenced by the different cross-section [6-8]. The standard deviation of the counterfeit group was very high, and the NCF was very low for some samples, which could indicate unstandardized or substandard manufacturing processes.

The devices used in dentistry directly affect the health of the patients. These individuals rely on dentists to provide the best care possible, and dentists must be able to trust the dental materials that they use. In recent years, a number of events have indicated that unprincipled manufacturers bring either illegal or counterfeit dental materials to the medical instrument market [4]. Additionally, there is a surprising lack of published information on this subject, possibly due to the difficulties in identifying counterfeit products.

Dentists should purchase safe, original dental products, either directly from the manufacturers or through their authorized distributors and dealers. There are several ways to identify possibly gray-market, imitation, or counterfeit dental supplies, including unusual low prices, unknown distributor names, and suspicious packaging [9].

## Conclusion

Original rotary instruments showed superior cyclic fatigue resistance when compared to counterfeit instruments. Clinicians should be careful not to purchase imitation dental products.

## Competing interests

The authors declare that they have no competing interests

## Authors’ contributions

HE participated in the design of the study and performed the statistical analysis. HE and IDC conceived of the study, and participated in its design and coordination and HA and EA helped to draft the manuscript. All authors read and approved the final manuscript.

## References

[B1] Sattapan B Nervo GJ Palamara JE Messer HH Defects in rotary nickel-titanium files after clinical use J Endod 2000 26 161 165 10.1097/00004770-200003000-00008 11199711

[B2] Berutti E Chiandussi G Paolino DS Scotti N Cantatore G Castellucci A Pasqualini D Canal shaping with WaveOne Primary reciprocating files and ProTaper system: a comparative study J Endod 2012 38 505 509 10.1016/j.joen.2011.12.040 22414838

[B3] Peters OA Barbakow F Dynamic torque and apical forces of ProFile.04 rotary instruments during preparation of curved canals Int Endod J 2002 35 379 389 10.1046/j.0143-2885.2001.00494.x 12059940

[B4] de Brujin ACP de Vries CGJCA Hermsen HPH Counterfeit medical devices : A risk indication RIVM 2009 Letter 360060001 1 20

[B5] Gambarini G Grande NM Plotino G Somma F Garala M De Luca M Testarelli L Fatigue resistance of engine-driven rotary nickel-titanium instruments produced by new manufacturing methods J Endod 2008 34 1003 1005 10.1016/j.joen.2008.05.007 18634935

[B6] Pedulla E Grande NM Plotino G Palermo F Gambarini G Rapisarda E Cyclic fatigue resistance of two reciprocating nickel-titanium instruments after immersion in sodium hypochlorite Int Endod J 2013 46 155 159 10.1111/j.1365-2591.2012.02100.x 22831397

[B7] Capar ID Ertas H Arslan H Comparison of cyclic fatigue resistance of novel nickel-titanium rotary instruments Aust Endod Jdoi:10.1111/aej.12067 10.1111/aej.12067 24697976

[B8] Capar ID Ertas H Arslan H Comparison of cyclic fatigue resistance of nickel-titanium coronal flaring instruments J Endoddoi:10.1016/j.joen.2013.12.031 10.1016/j.joen.2013.12.031 25069929

[B9] Christensen GJ Are you using “gray-market” or counterfeit dental products? J Am Dent Assoc 2010 141 712 715 10.14219/jada.archive.2010.0262 20516104

